# Data on seed germination using LED versus fluorescent light under growth chamber conditions

**DOI:** 10.1016/j.dib.2018.05.040

**Published:** 2018-05-12

**Authors:** Rabab Sanoubar, Roberta Calone, Enrico Noli, Lorenzo Barbanti

**Affiliations:** Department of Agricultural and Food Sciences (DISTAL), University of Bologna, Viale Fanin 44, 40127 Bologna, Italy

## Abstract

The present investigation attempted to assess the influence of two light sources, LED versus fluorescent light, on seed germination of nine aromatic species belonging to the genus *Artemisia*, *Atriplex*, *Chenopodium*, *Salicornia*, *Sanguisorba*, *Portulaca* and *Rosmarinus*. Pre-germination test was carried out in petri dishes, evidencing the need to overcome seed dormancy through cold stratification in *Salicornia europaea*. Thereafter, seeds were germinated in small trays with peat moss substrate in two growth chambers illuminated with either LED or fluorescent light featuring similar photosynthetic photon flux density. Germination lasted 20 days, during which time five indexes of germination performance (germination percentage, speed of germination, germination energy, germination rate index, and mean daily germination) were evaluated. At the end, shoot length and seedling fresh weight were assessed as early growth traits. Data are made available to allow critical evaluation of experimental outcome.

**Specifications Table**

**Table:** 

Subject area	Seed germination
More specific subject area	Comparing the germination performance of some aromatic seeds under LED versus fluorescent light.
Type of data	Text file, table and figures
How data was acquired	Seed germination procedure worksheet: petri dishes with continual moistening of filter paper in controlled germination chambers were used for pre-germination, while small trays filled with peat moss were used for seed germination and seedling development.
Data format	Analyzed using CoStat software
Experimental factors	Two combined factors potentially affecting seed germination: light source (LED versus fluorescent light) and seed species (nine).
Experimental features	2-Way Completely Randomized Design
Data source location	*Bologna, Italy,* Latitude: 44° 29′ 22.79′′ N, Longitude: 11° 20′ 20.40′′ E.
Data accessibility	Relevant data reported in this article

**Value of the data**

• LEDs were more efficient than fluorescent lamps in stimulating both seed germination and shoot growth.
• LED light compared to the traditional fluorescent lamps could be an alternative source of lighting particularly at the seed germination stage.
• The superiority of most germination parameters shown under LED lighting could be the basis for further investigation regarding the vegetative growth phase and the effect of LED light on essential oil composition of these aromatic plants.

## 1 Data

Data describe the performance of nine aromatic seed species under two light sources, Light-Emitting Diode (LED) and fluorescent light. Pre-germination was carried out in germination chambers for 18 days to assess the occurrence of seed dormancy in the nine-investigated species, and the need to overcome it through appropriate treatments. After performing such treatment on dormant seeds, the main germination treatment took place in growth chambers under LED vs. fluorescent lamps. Several germination traits were assessed for 20 days, including germination percentage (GP), speed of germination (SG), mean daily germination (MDG), germination energy (GE), and germination rate index (GRI) (Fig. 2). On the last day of the trial (20th day), seedling growth was assessed in terms of shoot length and seedling fresh weight (Fig. 3).

## 2 Experimental design, materials and methods

### 2.1 Plant species

Seeds of some aromatic plants such as *Artemisia absinthium, Artemisia vulgaris, Atriplex halimus, Atriplex hortensis* cv. ‘Plum Copper’*, Chenopodium quinoa* cv. ‘Cherry Vanilla’, *Sanguisorba minor*, *Portulaca oleracea*, *Rosmarinus officinalis* and *Salicornia europaea* were purchased from B & T World Seeds Company (http://b-and-t-world-seeds.com/homepage.htm). Pre-germination test was first carried out in germination chambers to assess the need of overcoming dormancy. Subsequently, two growth chambers featuring different light sources, LED vs. fluorescent lamps, were used to test seed germination and seedling development. The experiment was conducted at the Department of Agricultural and Food Sciences (DISTAL), University of Bologna, Italy.

### 2.2 Pre-germination treatment in germination chambers

As the germination can be highly affected by seed dormancy, the nine species were subjected to a pre-germination treatment in germination chambers. Healthy and uniform seeds of all species were surface-sterilized with solution of 3% sodium hypochlorite for two minutes. Seeds were later rinsed in deionized water for 5 min and were dried at room temperature. Two replicates of seeds were placed on filter paper moistened with 5 ml distilled water in 9-cm diameter petri dishes wrapped in transparent plastic sheets to prevent water evaporation. All petri dishes were kept in an incubator at 25 °C, 70–80% relative humidity, and 16/8 h light/dark period. The filter paper was controlled and repeatedly damped with fresh water according to need, in order to avoid excessive drying/moistening. A light paintbrush was used to remove initial signs of fungal growth, while old filter papers were replaced by new ones in case of massive fungal growth. The germination records were carried out during the 20-day test. The germinated seeds were picked out from the petri dishes after counting. Seeds were considered to be germinated when the emerging radicle was ≥ 2 mm long. In the pre-germination, we recorded acceptable germination rate for all species except *S. europaea* that always showed low germination rate (data not shown). Therefore, to ensure successful germination for *S. europaea,* it was necessary to overcome seed dormancy by the method of cold stratification [1], i.e. exposing the seeds to a period of cold. This consisted of placing 50 seeds of *S. europaea* on damp filter paper in a 9-cm petri dish wrapped in transparent plastic sheet for 30 days in a dark refrigerator at 6 °C.

### 2.3 Germination treatment and seedling development in growth chamber

Four replicates of seeds of all species, except *S. europaea*, were germinated directly on small trays filled with peat moss growing media. Conversely, *S. europaea* was subjected to cold stratification for one month as previously described, then the small seedlings were carefully transferred from petri dishes into the trays filled with peat moss. All seeds were germinated in two growth chambers: one of them was equipped with LED lighting systems, while the other with fluorescent lights. Both growth chambers were set at 22 ± 1 °C, 70% air humidity and 16/8 h light/dark period. The seeds were supplied daily with distilled water during the germination trial that lasted 20 days. Seeds were considered to be germinated when the emerging radicle was ≥ 2 mm long. The number of germinated seeds was recorded during the 20-day test. On the last day of the trial (20th day), two growth traits, shoot length and seedling fresh weight, were measured using five random seedlings from each replicate.

### 2.4 Lighting systems in growth chambers

**Fig. 1:**
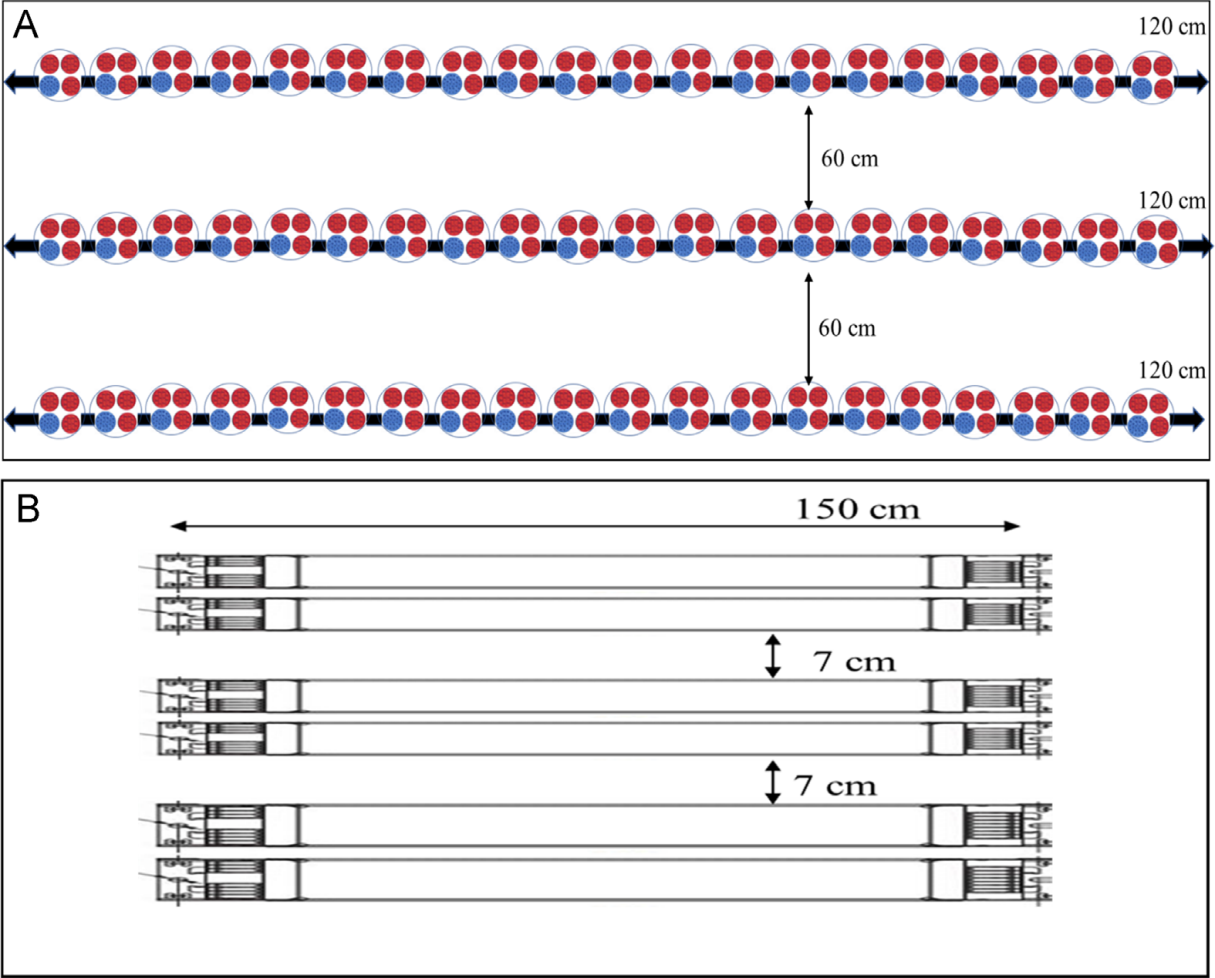
Sketch of the two growth chambers: A, illuminated by LED light system; B, illuminated by fluorescent lights.

Two different lighting systems were used in this experiment. One growth chamber was equipped with artificial LED lamps. The LED lights were composed of three lines of 120 cm length, while the distance between lines was 60 cm (Fig. 1A). Each line was composed of 80 diode lamps, where every four diode lamps were grouped together emitting light of two colors: three diodes emitted red light (655 nm) and one blue light (456 nm) under photosynthetic photon flux density (PPFD) of 200 μmol m^−2^ s^−^^1^. This level of PPFD was obtained by placing plants at 180 cm distance from the light source. PPFD was measured at the top of the plants using a quantum sensor (SKIE SKP 215 sensors, used with SKP 200 display meter).

The other growth chamber was illuminated using traditional fluorescent lights as a control (MASTER TL-D 90 De Luxe 58 W/950, Philips, Amsterdam, The Netherlands) (Table 1). The growth chamber was divided into several shelves. In each shelf the lighting system was a set of 6 fluorescent lamps of 150 cm length; they were aligned horizontally, every two lamps were grouped together and the distance between light groups was 7 cm (Fig. 1B). The distance between the light source and the shelf was 40 cm. PPFD measured at the top of the plants was 150 μmol m^−^^2^ s^−^^1^, i.e. quite similar to that of LED lights.

**Table 1:** Light technical properties of fluorescent lamps.

Correlated colour temperature	5300 K
Luminous efficacy	77.8 lm/W
Power	58.5 W
Lamp current	0.670 A
Colour rendering index	92 *R*_a_

### 2.5 Methods of calculation of germination parameters

Germination parameters were assessed as follows:

1. Germination Percentage (GP) [2]
  (1) $$\mathrm{GP}=\frac{\mathrm{Total}\;\mathrm{number}\;\mathrm{of}\;\mathrm{germinated}\;\mathrm{seeds}}{\mathrm{Total}\;\mathrm{number}\;\mathrm{of}\;\mathrm{seeds}\;\mathrm{per}\;\mathrm{assay}}*100$$
2. Speed of Germination (SG) [3]
  (2) $$\mathrm{SG}=\frac{n_{1}}{d_{1}}+\frac{n_{2}}{d_{2}}+\frac{n_{3}}{d_{3}}+\ldots \frac{n_{i}}{d_{i}}$$
  where *n* is the number of germinated seeds and *d* is the number of days
3. Mean Daily Germination (MDG) [4]
  (3) $$\mathrm{MDG}\;=\;\frac{\mathrm{Total}\;\mathrm{number}\;\mathrm{of}\;\mathrm{germinated}\;\;\mathrm{seeds}}{\mathrm{Total}\;\mathrm{number}\;\mathrm{of}\;\mathrm{days}}$$
4. Germination Energy (GE) [5]
  (4) $$\mathrm{GE}\;=\frac{\mathrm{Percentage}\;\mathrm{of}\;\mathrm{germinated}\;\mathrm{seeds}\;\mathrm{at}\;\mathrm{the}\;\mathrm{starting}\;\mathrm{day}\;\mathrm{of}\;\mathrm{germination}}{\mathrm{Total}\;\mathrm{number}\;\mathrm{of}\;\mathrm{seeds}\;\;\mathrm{per}\;\mathrm{assay}}$$
5. Germination Rate Index (GRI) [6]
  (5) $$\mathrm{GRI}\;=\;\frac{{\mathrm{GP}}_{1}}{d_{1}}+\frac{{\mathrm{GP}}_{2}}{d_{2}}+\frac{{\mathrm{GP}}_{3}}{d_{3}}+\ldots \frac{{\mathrm{GP}}_{i}}{d_{i}}$$
  where GP_1_ is the germination percentage on day 1, etc.

### 2.6 Statistical analysis

**Fig. 2:**
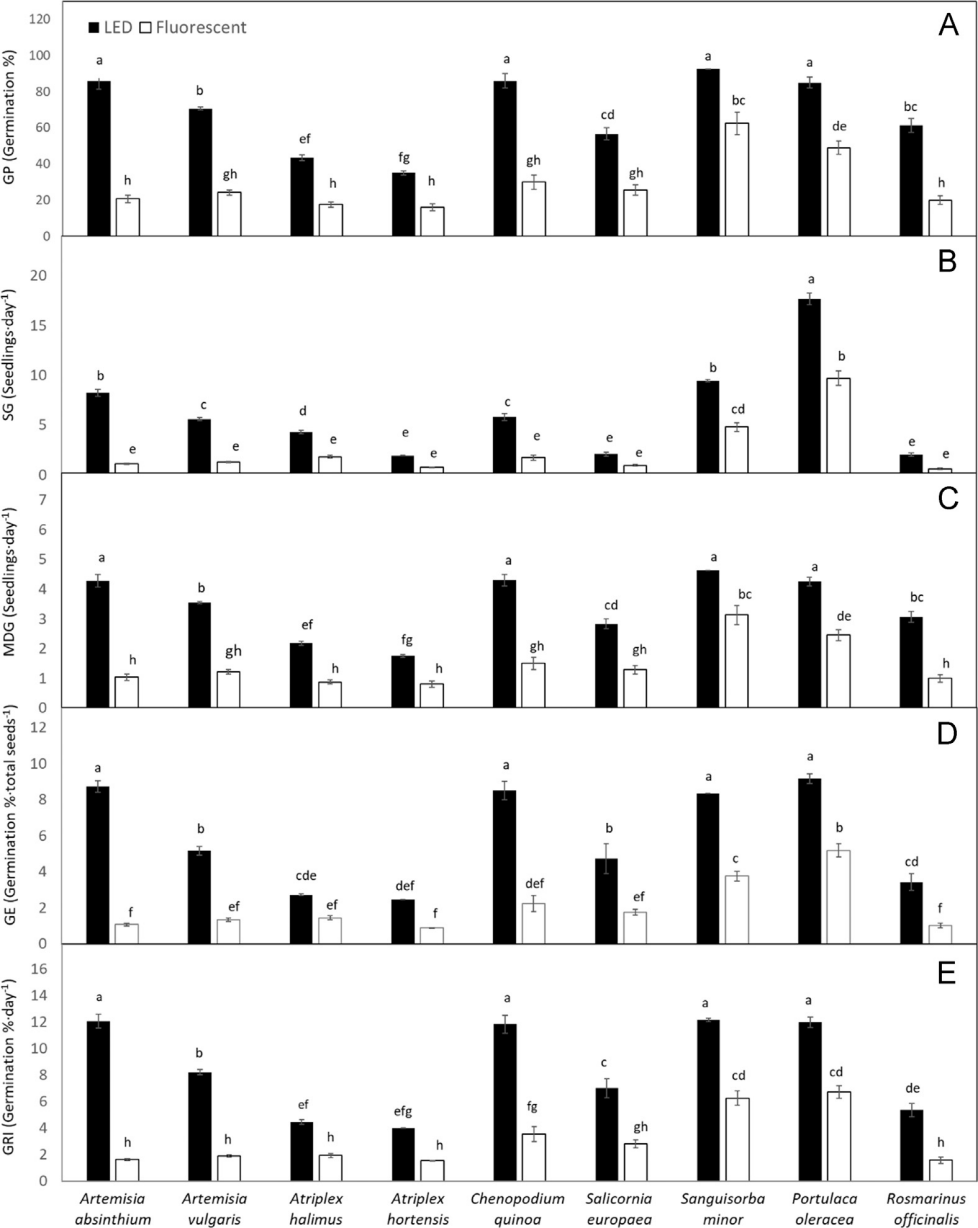
A, Germination percentage (GP); B, Speed of Germination (SG); C, Mean Daily Germination (MDG); D, Germination Energy (GE); E, Germination Rate Index (GRI) across some aromatic seed species as affected by LED and fluorescent lights. Mean values. Different letters indicate significant differences at *P* ≤ 0.05 (Tukey׳s test).

**Fig. 3:**
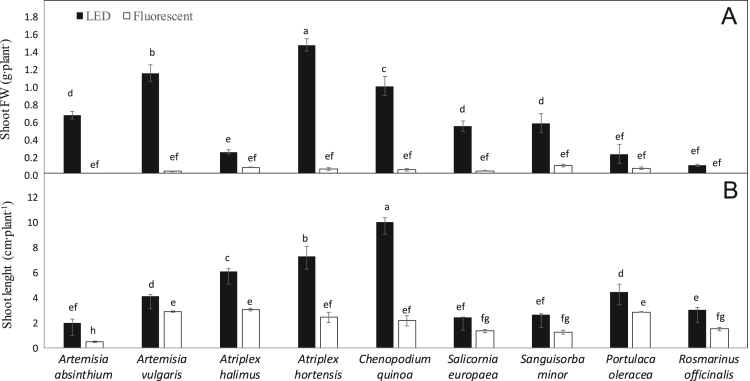
A, Shoot fresh weight; B, Shoot length across some aromatic seed species as affected by LED and fluorescent lights. Mean values. Different letters indicate significant differences at *P* ≤ 0.05 (Tukey׳s test).

The experiment was set up with a completely randomized design (CRD) with two replicates repeated twice in time, to reduce the experimental error. The data generated were submitted to *two-way ANOVA* using the statistical package CoStat, 6,4. Significant Species × Light Sources interactions at *P* ≤ 0.05 using Tukey׳s test are displayed in Fig. 2, Fig. 3.
